# Interferon-Tau Attenuates Uptake of Nanoparticles and Secretion of Interleukin-1β in Macrophages

**DOI:** 10.1371/journal.pone.0113974

**Published:** 2014-12-08

**Authors:** Kyoko Hara, Koumei Shirasuna, Fumitake Usui, Tadayoshi Karasawa, Yoshiko Mizushina, Hiroaki Kimura, Akira Kawashima, Akihide Ohkuchi, Shuichi Matsuyama, Koji Kimura, Masafumi Takahashi

**Affiliations:** 1 Division of Inflammation Research, Center for Molecular Medicine, Jichi Medical University, Shimotsuke, Tochigi, Japan; 2 Laboratory of Animal Reproduction, Department of Agriculture, Tokyo University of Agriculture, Atsugi, Kanagawa, Japan; 3 Department of Obstetrics and Gynecology, Jichi Medical University, Shimotsuke, Tochigi, Japan; 4 Animal Feeding and Management Research Division, National Institute of Livestock and Grassland Science, Nasushiobara, Tochigi, Japan; Virginia Tech University, United States of America

## Abstract

**Background:**

Type I interferons (IFNs), including IFN-alpha (IFNA) and IFN-beta (IFNB), have anti-inflammatory properties and are used to treat patients with autoimmune and inflammatory disorders. However, little is known of the role of IFN-tau (IFNT), a type I IFN produced by ruminant animals for inflammation. Because IFNB has recently been shown to inhibit nucleotide-binding oligomerization domain-like receptor, pyrin domain-containing 3 (NLRP3) inflammasome activation and subsequent secretion of the potent inflammatory cytokine interleukin (IL)-1β, we examined the effects of ruminant IFNT on NLRP3 inflammasome-mediated IL-1β secretion in human THP-1 macrophages.

**Methods and Results:**

IFNT dose-dependently inhibited IL-1β secretion induced by nano-silica, a well-known activators of NLRP3 inflammasomes, in human macrophages primed with lipopolysaccharide (LPS, TLR4 agonist) and Pam3CSK4 (TLR1/2 agonist). IFNT also suppressed phagocytosis of nano-silica and reactive oxygen species (ROS) generation. Western blot analysis showed that IFNT inhibited both pro-IL-1β and mature IL-1β. In addition, real-time RT-PCR analysis showed that IFNT suppressed IL-1β mRNA expression induced by LPS and Pam3CSK4. Although nano-silica particles did not induce IL-10 secretion, IFNT induced IL-10 secretion in a dose-dependent manner. Furthermore, IFNT-suppressed IL-1β secretion was restored by anti-IL-10 neutralizing antibody.

**Conclusions:**

Ruminant IFNT inhibits NLRP3 inflammasome-driven IL-1β secretion in human macrophages via multiple pathways, including the uptake of nano-silica particles, generation of ROS, and IL-10-mediated inhibition of pro-IL-1β induction. It may be a therapeutic alternative to IFNA and IFNB.

## Introduction

Type I interferons (IFNs) including IFN alpha (IFNA), IFN beta (IFNB), and IFN omega (IFNW) play crucial roles in the first line of defense against viral infection, activation of innate immune and adaptive immune systems, and the pathogenesis of various diseases [1], [2]. All type I IFNs bind common receptors such as IFNA receptor 1 (IFNAR1) and IFNAR2, induce several common genes, and exert various biological functions. Furthermore, type I IFNs have been used for treating patients with cancer, chronic viral infections, and multiple sclerosis (MS) [2], [3]. However, severe adverse effects and potential toxicity, including inflammatory and autoimmune complications, limit the clinical use of type I IFNs.

Unlike IFNA and IFNB, the type I IFN-tau (IFNT) has unique properties that are not virus-inducible and is secreted by embryonic trophoblast cells during pregnancy in ruminants [4]. IFNT has sequence similarity (approximately ∼50% amino acid identity) and functional homology to IFNA in humans, mice, and rats [5]. Although IFNT is produced only in ruminants, it has broad cross-species activity in humans and mice, and functions in various cell types such as macrophages, lymphocytes, and epithelial cells [5]. Accordingly, IFNT has been shown to decrease fetal resorption and inhibit the occurrence of experimental allergic encephalomyelitis (EAE) in an animal model of MS, and ameliorates spontaneous autoimmune diabetes in mice [6]. In particular, IFNT reportedly has low cytotoxicity and few adverse effects, indicating therapeutic potential as an alternative to other type I IFNs [7]. Currently, several phase III clinical trials are being conducted with IFNT for the treatment of patients with MS [8].

Accumulating evidence indicates that inflammatory responses in the absence of pathogens, referred to as sterile inflammation, are mediated by the nucleotide-binding oligomerization domain-like receptor (NLR) family, pyrin domain-containing 3 (NLRP3) inflammasomes [9], [10], [11]. NLRP3 inflammasomes are large cytosolic protein complexes that regulate the secretion of the pivotal inflammatory cytokine interleukin (IL)- 1β [9], [10], [11], and they comprise 3 different proteins, namely: NLRP3, apoptosis-associated speck-like protein containing a caspase recruitment domain (ASC), and caspase-1 as an IL-1β-converting enzyme. Increasing body of evidence suggests that NLRP3 inflammasomes contribute to the development of infectious and sterile inflammatory diseases [11],[12],[13],[14],[15]. Our group also recently demonstrated the importance of NLRP3 inflammasomes in neointimal formation after vascular injury, atherosclerosis, myocardial ischemia-reperfusion injury, and chronic renal disease [16], [17], [18], [19]. Because anti-inflammatory properties are a prominent feature of type I IFNs, several studies have shown that IFNB inhibits IL-1β secretion and NLRP3 inflammasome activation [20], [21]. In addition, Inoue et al. [21] recently reported that IFNB therapy ameliorates the development of murine EAE by inhibiting NLRP3 inflammasomes. However, contrary to these reports, several investigations reported that type I IFN is required for the activation of the non-canonical NLRP3 inflammasomes [22], [23]. Therefore, the role of type I IFNs in NLPR3 inflammasomes is controversial, and especially the effect of IFNT on NLRP3 inflammasomes and subsequent IL-1β secretion remains unknown. In the present study, we investigated the effects of ruminant IFNT on NLRP3 inflammasome-mediated IL-1β secretion in THP-1 human macrophages and found that IFNT inhibits IL-1β secretion induced by nano-silica, a well-known danger signal that activates NLRP3 inflammasomes, through multiple pathways. In the present study, we identified novel roles of IFNT and assessed the therapeutic potential of IFNT.

## Materials and Methods

### Preparation of IFNs and cell culture

Recombinant bovine IFNT (rbIFNT) was produced by *Escherichia coli.* Briefly, bovine IFNT cDNA (bTP-509A, gifted by Dr. RM Roberts, University of Missouri, Columbia) was inserted into pET-21a (Invitrogen) *E.coli* expression vectors [4]. After purification of crude IFNT by HPLC, we confirmed low levels of endotoxin using the LAL Endotoxin assay kit (GenScript, Piscataway, NJ). The activity of IFNT was then determined by a viral resistance assay using bovine kidney MDBK cells and was found to be 59050 IU/mL (5.95×10^5^ IU/mg at 456 µM). Recombinant human IFNB (rhIFNB) was obtained from Peprotech (Rocky Hill, NJ).

Human THP-1 cell lines were obtained from American Type Culture Collection (ATCC, University Boulevard, Manassas, VA). THP-1 were cultured in RPMI-1640 (Life Technologies Corporation, Carlsbad, CA) supplemented with antibiotics and 10% fetal calf serum (Dainippon Pharmaceutical Company, Osaka, Japan), and were differentiated into macrophages following treatment with 200 nM phorbol 12-myristate 13-acetate (PMA, Sigma, St. Louis, MO) for 24 h. To detect IL-1β secretion, cells were washed twice in phosphate buffered saline and pretreated for 48 h with or without rbIFNT in an RPMI 1640 medium containing 0.1% bovine serum albumin. After priming with lipopolysaccharide as TLR4 lignad (LPS, 100 ng/mL, Sigma) for 3 h or Pam3CysSerLys4 as TLR1/2 ligand (Pam3CSK4, 300 ng/mL, Life Technologies, San Diego, CA) for 10 h to induce pro-IL-1β synthesis, THP-1 macrophages were treated with 30-nm nano-silica particles (Micromod Partikeltechnologie, GmbH, Rostock, Germany) or adenosine 3′-phosphate (ATP, Sigma). Fluorescein isothiocyanate-labeled nano-silica (Green nano-silica) was purchased from Micromod Partikeltechnologie. For blocking experiments, anti-human IL-10 neutralizing antibody (2 µg/mL, PetroTech, Rocky Hill, NJ) or control IgG (Vector Laboratories, Burlingame, CA) were pretreated with IFNT treatment. Murine peritoneal macrophages were isolated from C57BL/6J mice injected with thioglycolate as described previously [25].

### Determination of inflammatory cytokines

IL-1β, IL-1α, and IL-10 levels were determined using a human enzyme-linked immunosorbent assay (ELISA) kit (R&D Systems, Minneapolis, MN) according to the manufacturer's instructions.

### Lactate dehydrogenase (LDH) activity assay

Cytotoxicity was determined as LDH activity using cytotoxicity detection kit (Roche, Mannheim, Germany) according to the manufacturer's instructions.

### Western blot analysis

The expression of IL-1β in supernatants was analyzed using SDS-PAGE. After transfer onto PVDF membranes, non-specific antibody binding was blocked for 1 h at room temperature using 5% skim milk. Membranes were then incubated for 1 h at room temperature or 24 h at 4C with anti-human IL-1β antibody (R&D systems), anti-signal transducer and activator of transcription 3 (STAT3) antibody and anti-phospho-STAT3 antibody (Cell Signaling Technology, Inc., Boston, MA), anti-caspase-1 antibody (Cell Signaling) followed by incubation for 1 h with secondary antibody conjugated horseradish peroxidase (HRP; Jackson ImmunoResearch Laboratories, Inc., PA). Immunoreactive bands were visualized by Western BLoT Quant HRP Substrate (Takara Bio Inc., Shiga, Japan).

### Detection of reactive oxygen species (ROS) generation and F-actin formation

THP-1 macrophages were pretreated with or without rbIFNT for 48 h and stimulated with nano-silica at 37°C before harvest. ROS generation (H_2_O_2_) was determined using dichlorodihydrofluorescein (DCFDA; Wako Chemicals, Osaka, Japan).

To detect F-actin formation, cells were fixed and permeabilized using a FoxP3 staining buffer set (Miltenyi Biotec, Bergisch Gladbach, Germany), and were then stained with Anti-stain 488 fluorescent phalloidin (Cytoskelton, Inc., CO). Nuclei were stained with Hoechst 33342 (Immunochemistry Technologies, Bloomington, MN) and the fluorescence within cells was examined by flow cytometry (FACSCalibur; Becton Dickinson, Franklin Lakes, NJ) and analyzed using FlowJo software version 10 (Tree Star) or using confocal laser scanning microscopy (FV-10i; Olympus, Tokyo, Japan).

### Real-time RT-PCR

Total RNA was prepared using ISOGEN (Nippon Gene Company, Limited, Toyama, Japan) according to the manufacturer's instructions. Real-time RT-PCR was performed using the Thermal Cycler Dice Real Time System II (Takara Bio Incorporated, Shiga, Japan) to detect mRNA expressions of ISG15, IL-1β, IL-1α, NLRP3, ASC, CASPASE-1, macrophage receptor with collagenous structure (MARCO), CD36, scavenger receptor (SR)-B1, macrophage scavenger receptor 1 (MSR1), oxidized low-density lipoprotein receptor 1 (OLR1) and GAPDH. The following antisense and sense primers were used: *ISG15* (5′-TGT CCC TGA GCA GCT CCA TG-3′ and 5′-TGT CCT GCA GCG CCA CAC C-3′), *IL1β* (5′-TGA TGG CTT ATT ACA GTG GCA ATG-3′ and 5′-GTA GTG GTG GTG GGA GAT TCG-3′), *IL1α* (5′- TGA CTG CCC AAG ATG AAG ACC-3′ and 5′- TCC CAG AAG AAG AGG AGG TTG-3′), *NLRP3* (5′-GAG AGA CCT TTA TGA GAA AGC A-3′ and 5′-GCA TAT CAC AGT GGG ATT CGA A-3′), *ASC* (5′-AAC CCA AGC AAG ATG CGG AAG-3′ and 5′-TTA GGG CCT GGA GGA GCA AG-3′), *CASPASE1* (5′-GAA GCT CAA AGG ATATGG AAA CAA A-3′ and 5′-AAG ACG TGT GCG GCT TGA CT-3′), *MARCO* (5′-TGC TGG GTT ACT CCA AAG GA-3′ and 5′-CAG CCA GAT CTG CCC AGT-3′), *CD36* (5′-GAG AAC TGT TAT GGG GCT AT-3′ and 5′-TTC AAC TGG AGA GGC AAA GG-3′), *SR-B1* (5′-TGA TGA TGG AGA ATA AGC CCA T-3′ and 5′-TGA CCG GGT GGA TGT CCA GGA AC-3′), *MSR1* (5′- GCA GTG GGA TCA CTT TCA CAA-3′ and 5′- AGC TGT CAT TGA GCG AGC ATC-3′), *OLR1* (5′- TTG CCT GGG ATT AGT AGT GAC C-3′ and 5′- GCT TGC TCT TGT GTT AGG AGG T-3′), glyceraldehyde 3-phosphate dehydrogenase *(GAPDH)* (5′-AAA TGA GCC CCA GCC TTC T-3′ and 5′-AGG ATG TCA GCG GGA GCC GG-3′). Expression levels of each target gene were normalized to corresponding GAPDH threshold cycle (CT) values using the ΔΔ CT comparative method.

### siRNA knockdown for MARCO

Human MARCO-targeting siRNA oligonucleotides (siMARCO) were obtained from Sigma. The sequences of each siRNA oligonucleotide in this pool are as follows: *h-siMARCO*-1, 5′-GGU AGA CAA CUU CAC UCA TT-3′; *h-siMARCO*-2, 5′-CUC AGU GUC CGU CAG GAU UTT-3′ (MISSION siRNAs, SIGMA). Mission siRNA universal negative control (SIGMA) was used as a negative control siRNA (siN.C.). For MARCO knockdown experiments, cells were seeded in 24-well plates at a density of 2×10^5^ cells per well in the presence of 200 nM PMA and allowed to attach to culture dishes for 24 h. The differentiated cells were then transfected with a pooled combination of two siRNA oligonucleotides (33 nM) for 6 h using Lipofectamine 2000 (1.67 µl/mL) in 600 µL of OPTI-MEM I medium. The next day, cells were washed with OPTI-MEMI medium (Life Technologies). Forty-eight hours after transfection, cells were harvested for mRNA analysis or stimulated with nanosilica.

### Statistical analysis

Data are expressed as mean ± standard error of the mean (SEM). Differences between treatment groups were identified using unpaired *t*-tests. Multiple comparisons were performed using one-way analysis of variance (ANOVA) followed by Bonferroni multiple comparison tests. A *p* value of <0.05 was considered to be statistically significant.

## Results

### Inhibitory effects of rbIFNT on nano-silica-induced IL-1β secretion

**Figure 1:**
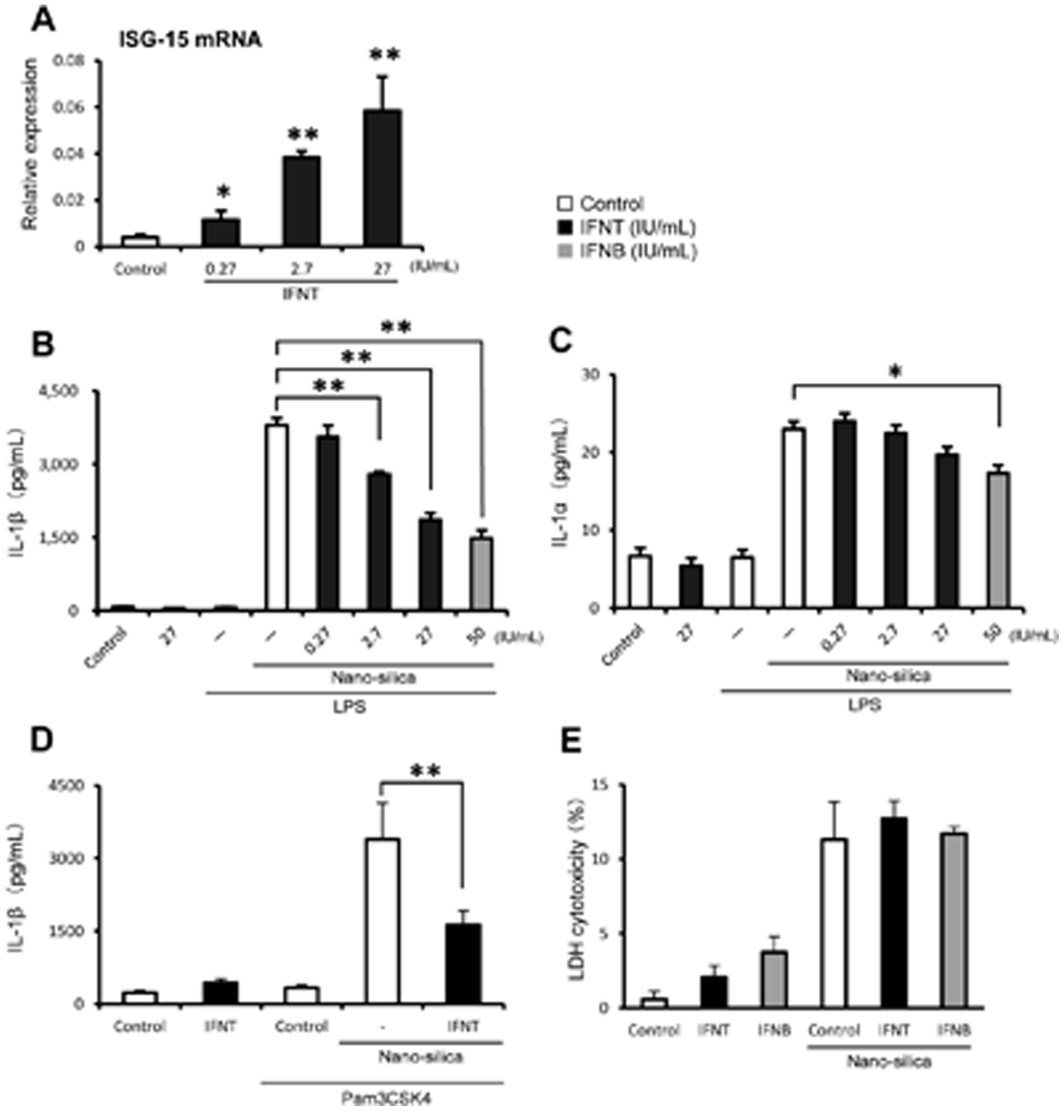
Effects of rbIFNT on ISG15 expression and inflammatory cytokine secretion. (A) THP-1 macrophages were incubated for 48 h with or without rbIFNT at the indicated concentrations. Subsequently, total RNA was extracted and analyzed by real-time RT-PCR for expression of ISG15 mRNA. (B–D) THP-1 macrophages were incubated for 48 h with rbIFNT or rhIFNB at the indicated concentrations. After priming with LPS (100 ng/mL) for 3 h or Pam3CSK4 (300 ng/ml) for 10 h, cells were treated with nano-silica particles (100 µg/mL) for 6 h. IL-1β (B and D) and IL-1α (C) levels in supernatants were then determined using ELISA. (E) LDH release from THP-1 macrophages treated with rbIFNT or rhIFNB. Data are expressed as means ±SEM (n = 3); Significant differences were identified using ANOVA; *p<0.05 and ** *p*<0.01.

Initially, we examined cross-species activities of rbIFNT in human THP-1 macrophages, and confirmed that rbIFNT treatment of THP-1 macrophages significantly and dose-dependently increased ISG15 mRNA expression, which is a well-known marker of IFNT responses (Fig. 1A). We also confirmed that rhIFNB increased ISG15 mRNA expression (data not shown). Because type I IFNs activate STAT3 [24], we examined whether IFNT can activate STAT3 in THP-1 macrophages. Similar to rhIFNB, rbIFNT also induced STAT3 phosphorylation in THP-1 macrophages (Fig. S1). Previously, we showed that nano-silica particles stimulated processing of pro-IL-1β into its mature form following activation of the NLRP3 inflammasome [25]. Thus, in the present study we investigated the effect of rbIFNT on nano-silica-induced IL-1β secretion. After priming with low-dose LPS (TLR4 agonist) to induce pro-IL-1β synthesis, cells were stimulated with 100 µg/mL nano-silica particles. Nano-silica particles drastically stimulated IL-1β secretion in the supernatant (Fig. 1B). Pretreatment with rbIFNT suppressed IL-1β secretion in a dose-dependent manner. Consistent with a previous study [20], IFNB also inhibited nano-silica-induced IL-1β secretion (Fig. 1B). We next examined the inhibitory effect of IFNs using another NLRP3 inflammasome activator ATP [26]. Pretreatment with rbIFNT significantly suppressed ATP-induced IL-1β secretion, but this inhibition was less compared with rhIFNB (Fig. S2A). To investigate whether IFNT can function macrophages derived from other species, we used murine peritoneal cavity macrophages. Treatment with rbIFNT failed to inhibit nano-silica-induced IL-1β secretion, indicating no cross-reactivity of rbIFNT to murine macrophages (Fig. S2B). We also examine the effect of IFNT using Pam3CSK4 (TLR1/2 agonist) as a priming stimulus and found that rbIFNT (27 IU/mL) significantly suppressed nano-silica-induced IL-1β secretion even in the Pam3CSK4 priming condition (Fig. 1D). Although rhIFNB significantly inhibited nano-silica-induced IL-1α secretion, rbIFNT gradually, not significantly, inhibited it (Fig. 1C). Because we previously showed that nano-silica particles induced cell death [25], cytotoxicity was assessed by measurement of LDH activity. Treatment with rbIFNT (27 IU/mL) and rhIFNB (50 IU/mL) had no effect on nano-silica-induced cell death (Fig. 1E).

### Effects of rbIFNT on phagocytosis of nano-silica and actin polymerization

**Figure 2:**
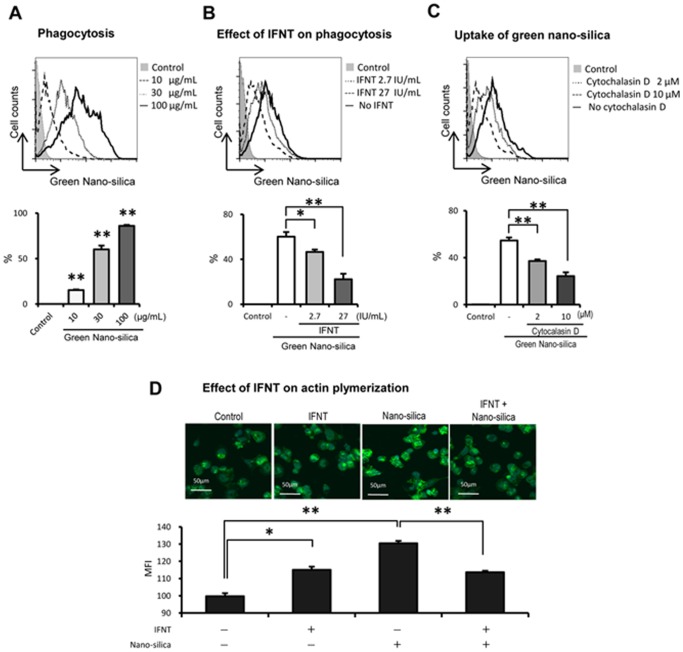
Effects of rbIFNT on nano-silica uptakes and actin polymerization. (A) THP-1 macrophages were treated with Green nano-silica for 6 h at the indicated concentrations. (B) THP-1 macrophages were incubated for 48 h with or without rbIFNT (2.7 and 27 IU/mL), and were then treated with Green nano-silica particles (30 µg/mL) for 6 h. After washing cells, uptake of Green nano-silica particles was analyzed by flow cytometry. (C) THP-1 macrophages were incubated for 1 h with or without cytochalasin D and were treated with Green nano-silica particles (30 µg/mL) for 6 h. After washing cells, uptake of Green nano-silica particles was analyzed by flow cytometry. Representative flow cytometry plots are presented. (D) THP-1 macrophages were incubated for 48 h with or without rbIFNT (27 IU/mL), and were then treated with nano-silica particles (30 µg/mL) for 6 h. Cells were stained with fluorescent-conjugated phalloidin and analyzed by flow cytometry. Nuclei were co-stained with Hoechst 33342. Representative confocal microscopic images of the F-actin assembly are presented. Quantitative analyses were performed and data are expressed as means ±SEM (n = 3); Significant differences were identified using ANOVA; *p<0.05 and ** *p*<0.01.

Nano-silica particles trigger an inflammation following endocytosis into the target cells. To assess regulation of phagocytosis of nano-silica by rbIFNT, we used Green nano-silica particles and performed flow cytometry analyses. Dose-dependent incorporation of Green nano-silica particles into THP-1 macrophages (Fig. 2A) was significantly inhibited in cells that were pretreated with rbIFNT (Fig. 2B).

To further investigate the inhibitory effect of rbIFNT on nano-silica uptake in THP-1 macrophages, we used the actin-disrupting agent cytochalasin D to block phagocytosis. Pretreatment with cytochalasin D clearly suppressed the uptake of nano-silica in a dose-dependent manner (Fig. 2C). Accordingly, F-actin levels in THP-1 macrophages, which were determined using fluorescent-conjugated phalloidin, were considerably increased in response to nano-silica particles. This increase in F-actin levels was significantly inhibited in rbIFNT-pretreated cells (Fig. 2D), indicating the involvement of actin polymerization in rbIFNT-inhibited uptake of nano-silica particles. F-actin levels were also increased following pretreatment with rbIFNT alone. Confocal laser scanning microscopic analyses confirmed the inhibitory effects of rbIFNT on nano-silica-induced actin polymerization (Fig. 2D).

### Effects of rbIFNT on scavenger receptors

**Figure 3:**
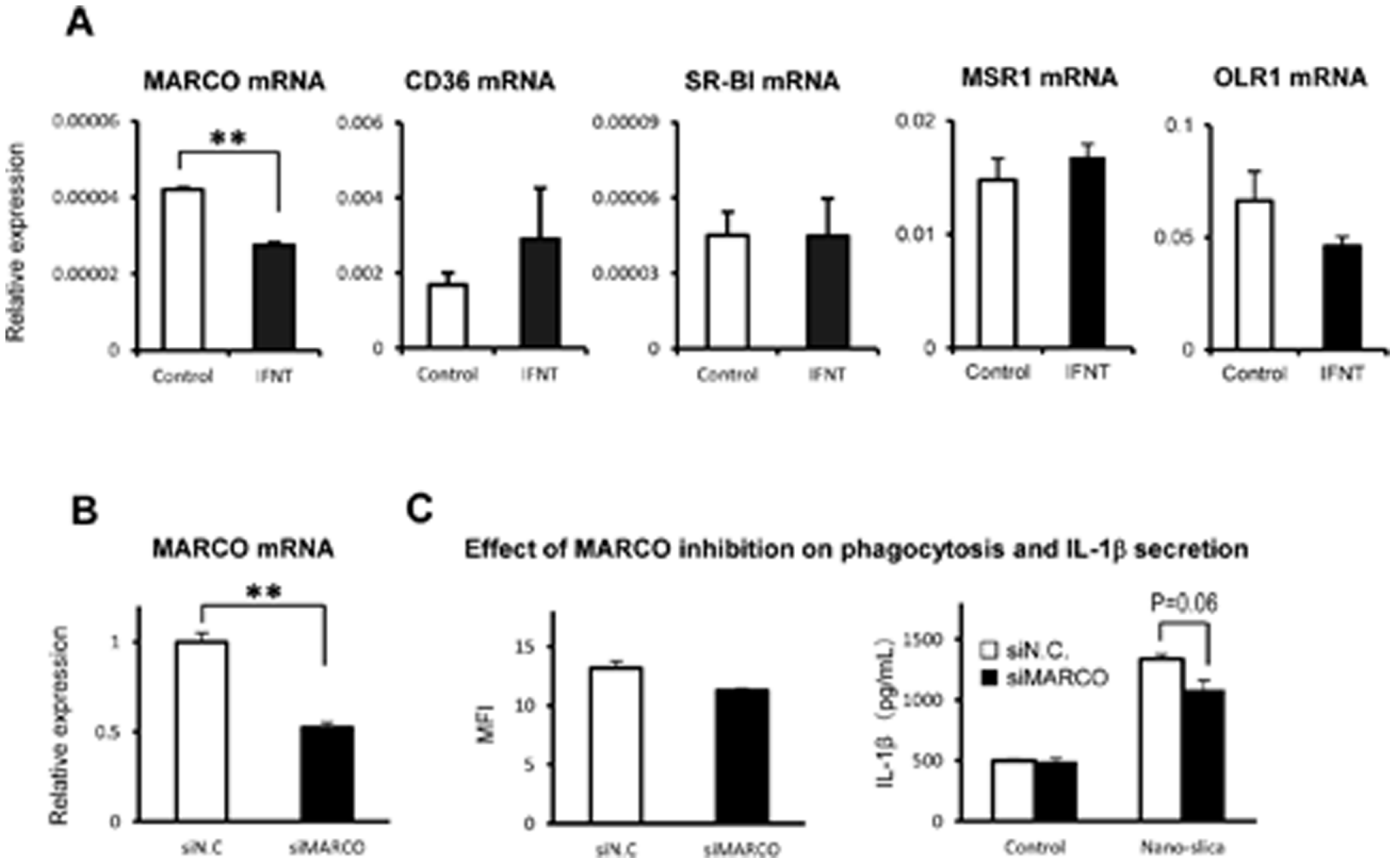
Effects of rbIFNT on expression of scavenger receptors and the functional analysis of MARCO by siRNA knockdown. (A) THP-1 macrophages were incubated for 48 h with or without rbIFNT (27 IU/mL) and total RNA was then extracted and analyzed by real-time RT-PCR for MARCO, CD36, SR-BI, MSR1 and OLR1 mRNA. (B) THP-1 macrophages were transfected with MARCO siRNA (siMARCO) or control siRNA (siN.C.) and analyzed MARCO mRNA expression by real-time RT-PCR (C). THP-1 macrophages transfected with MARCO siRNA or control siRNA were incubated with green nano-silica particles (30 µg/mL) for 6 h. After washing cells, uptake of green nano-silica particles was analyzed the amount of nano-silica (mean fluorescence intensity; MFI) by flow cytometry. After nano-silica treatment, IL-1β levels in supernatants were determined using ELISA. Data are expressed as means ±SEM (n = 3–4); Significant differences were identified using t-test; ** *p*<0.01 vs. control.

Recently, Kanno et al. [27] reported that MARCO, which is a macrophage-expressed SR, plays a role in clearing nanoparticles such as TiO_2_ and Fe_2_O_3_. Therefore, we examined the effect of rbIFNT on the expression of MARCO and other SRs and found that rbIFNT significantly inhibited expression of MARCO, but not CD36, SR-BI, MSR1, and OLR1 mRNA in THP-1 macrophages (Fig. 3A).

### Functional analysis of MARCO on phagocytosis and IL-1β secretion

Because IFNT suppressed MARCO expression together with inhibition of phagocytosis of nano-silica particles, we hypothesized that IFNT decreases the uptake of nano-silica particles via inhibition of MARCO expression. To test this hypothesis, we used siRNA technique. As expected, THP-1 macrophages transfected with MARCO siRNA decreased almost half of MARCO mRNA expression, compared with control siRNA (Fig. 3B). However, the knockdown of MARCO did not affect the uptake of Green nano-silica particles (Fig. 3C). The MARCO knockdown tended to decrease nano-silica-induced IL-1β secretion, compared with control siRNA (Fig. 3C).

### Effects of rbIFNT on ROS production

**Figure 4:**
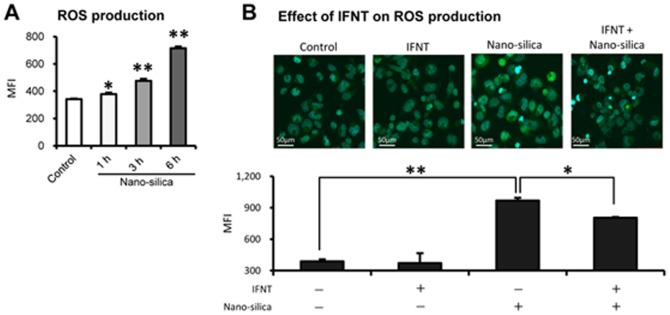
Effects of rbIFNT on ROS generation. (A) THP-1 macrophages were treated with nano-silica (100 µg/mL) for the indicated periods. ROS generation was assessed using DCFDA and flow cytometry. (B) THP-1 macrophages were incubated for 48 h with or without rbIFNT (27 U/mL) and were treated with nano-silica particles (100 µg/mL) for 6 h. ROS generation was assessed using DCFDA by flow cytometry. Quantitative analyses were performed and data are expressed as means ±SEM (n = 3); Significant differences were identified using ANOVA; *p<0.05 and ** *p*<0.01. Representative confocal microscopic images of ROS production in THP-1 macrophages; Nuclei were co-stained with Hoechst 33342.

Because ROS are major activators of the NLRP3 inflammasomes [21], we examined the effect of rbIFNT on ROS generation by flow cytometry. ROS generation was determined using the DCFDA dye for H_2_O_2_, which was clearly detected following treatment with nano-silica (100 µg/mL) in a time-dependent manner in THP-1 macrophages (Fig. 4A). Furthermore, the rbIFNT pretreatment partially but significantly diminished nano-silica-induced ROS generation, although it did not affect basal ROS levels (Fig. 4B).

### Mechanism of rbIFNT-suppressed IL-1β secretion

**Figure 5:**
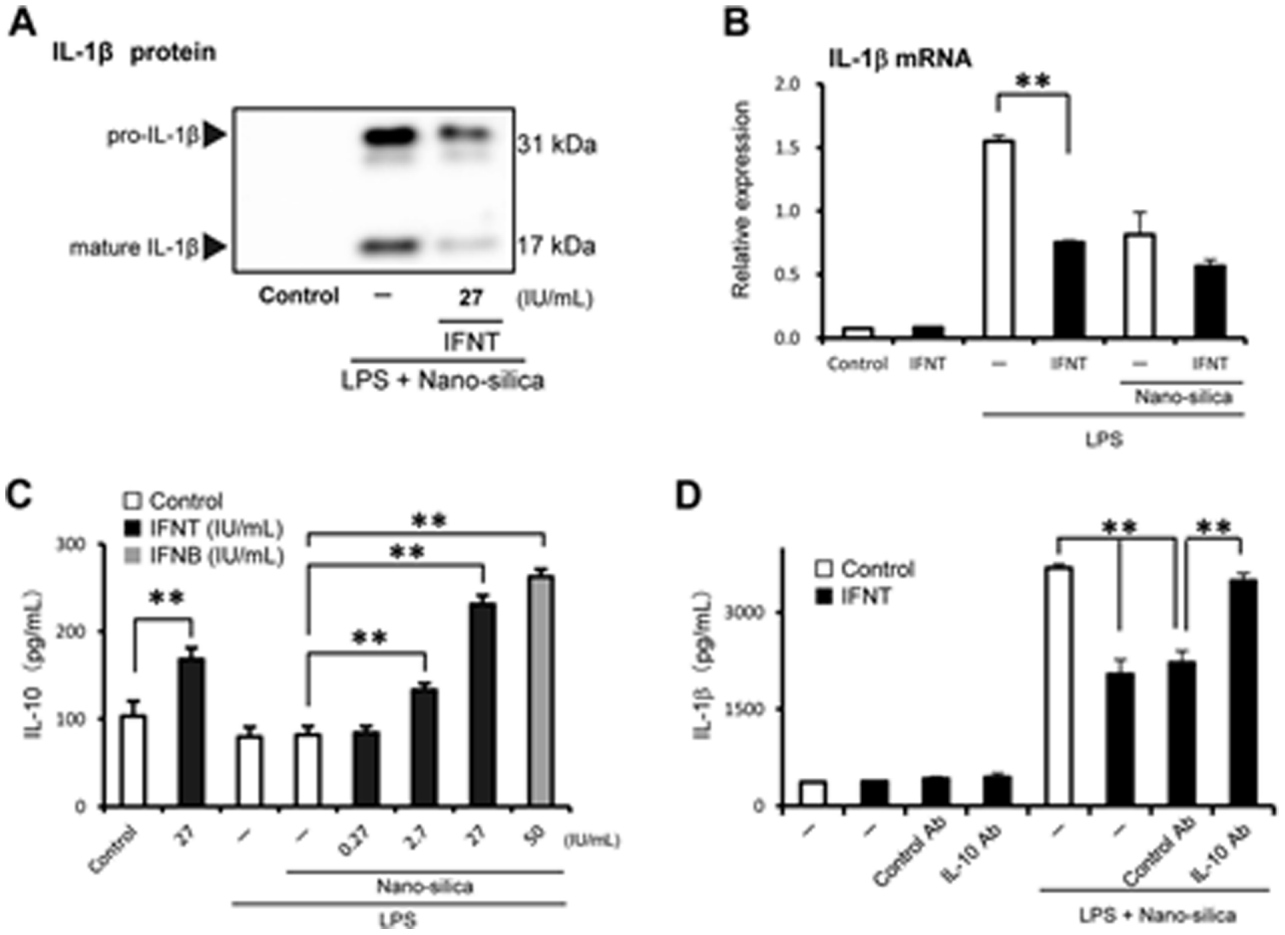
Effects of rbIFNT on IL-1β production and the role of IL-10. (A) THP-1 macrophages were incubated for 48 h with or without rbIFNT. After priming with LPS (100 ng/mL) for 3 h, cells were treated with nano-silica particles (100 µg/mL) for 6 h. Protein levels of pro-IL-1β and mature IL-1β in supernatants were detected by Western blot analyses. Representative photographs are shown. (B) Subsequently, total RNA was extracted and analyzed by real-time RT-PCR for expression of IL-1β mRNA. (C) IL-10 levels in supernatants were then determined using ELISA. (D) THP-1 macrophages were incubated with or without rbIFNT (27 IU/mL) in the presence of antibody against human IL-10 or control IgG, and then treated with LPS and nano-silica. IL-1β levels in supernatants were then determined using ELISA. Data are expressed as means ±SEM (n = 3); Significant differences were identified using ANOVA; ** *p*<0.01.

It is well accepted that 2 signals are required to induce mature IL-1β secretion [28]. First signal is provided by the TLR pathway to induce pro-IL-1β synthesis, and second signal is the inflammasome pathway, which processes pro-IL-1β to its biologically active mature form. Western blot analysis showed that rbIFNT inhibited both pro-IL-1β and mature IL-1β (Fig. 5A), suggesting inhibitory effects of IFNT on IL-1β induction at transcriptional and processing levels. Real-time RT-PCR analysis showed that rbIFNT clearly suppressed IL-1β mRNA expression induced by LPS and Pam3CSK4 at the priming condition (Fig. 5B and Fig. S3A). In addition, we assessed the effect of rbIFNT on the expression of the NLRP3 inflammasome-related genes and processing of caspase-1. Treatment with rbIFNT failed to affect NLRP3, ASC, or caspase-1 mRNA expression (Fig. S2C–E) and caspase-1 cleavage in THP-1 macrophages (Fig S2F). Supporting the data on IL-1α secretion (Fig. 1C), IL-1α mRNA expression induced by LPS priming was not inhibited by rbIFNT (Fig. S3B).

Since a previous investigation indicates that IFNB attenuates NLRP3 inflammasome-driven IL-1β secretion via, at least in part, anti-inflammatory cytokine IL-10 [20], [21], we finally investigated the role of IL-10 in IFNT-suppressed IL-1β secretion. Nano-silica particles did not induce IL-10 secretion in primed and nonprimed THP-1 macrophages, whereas rbIFNT dose-dependently induced IL-10 secretion, suggesting that IFNT induces IL-10 secretion independent of the nano-silica-induced NLRP3 inflammasomes (Fig. 5C). Furthermore, rbIFNT-suppressed IL-1β secretion was restored by anti-IL-10 neutralizing antibody (Fig. 5D).

## Discussion

Inflammation is a pivotal part of the innate immune response to pathogens, foreign substances, and endogenous danger signals released from injured tissues, and it is a hallmark of various diseases. Although appropriate inflammation contributes to protection from invading pathogens and injurious stimuli, as well as initiating tissue healing and repair, excessive inflammation is deleterious and leads to inflammatory diseases. In the present study, we found that ruminant IFNT suppresses IL-1β secretion in response to nano-silica particles, a well-known NLRP3 inflammasome activator, in TLR-mediated priming conditions in human macrophages. IFNT contributes to multiple pathways that inhibit the induction of pro-IL-1β, nano-silica uptake and ROS generation and promote the secretion of the anti-inflammatory cytokine IL-10, thereby inhibiting subsequent IL-1β secretion. Because the toxicity and adverse effects of IFNT are minimal in humans compared with other type I IFNs, the present data indicate that IFNT has potential as an alternative therapy for inflammatory and autoimmune diseases currently treated with IFNA and IFNB.

Type I IFNs were originally found to activate antiviral immune responses. Recently, anti-inflammatory properties of type I IFNs have received considerable attention, and IFNB has been introduced as a first-line therapy for patients with MS [3]. In addition, Inoue et al. [21] recently showed that IFNB clearly ameliorates EAE by inhibiting NLRP3 inflammasome activation in a murine model. They further showed that IFNB decreased the generation of mitochondrial ROS, a major trigger and an upstream regulator of NLRP3 inflammasome activation, in macrophages [21]. Roles of type I IFNs have also been shown in familial Mediterranean fever, an autoinflammatory syndrome associated with NLRP3 dysregulation and aberrant IL-1β secretion [29]. In agreement, Guarda et al. [20] demonstrated that type I IFNs inhibit inflammasome activation and IL-1β secretion through STAT1-dependent mechanisms, indicating that therapeutic potential of type I IFNs for various inflammatory diseases that are mediated by NLRP3 inflammasomes. Similar to IFNB, we observed that IFNT significantly suppressed nano-silica-induced IL-1β secretion and ROS generation in macrophages. Interestingly, however, IFNT also significantly inhibited pro-IL-1β induction induced by TLR ligands and did not affect nano-silica-induced activation of caspase-1. Because it was proposed that mature IL-1β secretion needs 2 signals; (1) TLR-mediated pro-IL-1β induction and (2) the inflammasome-mediated pro-IL-1β processing. Therefore, we assume that IFNT suppressed NLRP3 inflammasome-derived IL-1β secretion mainly through inhibiting pro-IL-1β induction in macrophages.

Interleukin-10 is a pivotal anti-inflammatory cytokine and appears to be a key mediator for preventing inflammatory diseases [30], [31]. Guarda et al. [20] previously reported that IFNB attenuates NLRP3 inflammasome-driven IL-1β secretion via mechanisms involving (1) induction of IL-10 secretion, which acts as an autocrine and/or paracrine signal that inhibits the synthesis of pro-IL-1β and (2) by directly inhibiting NLRP3 inflammasome activation. In the present study, we observed secretion of IL-10 in IFNT-treated macrophages together with suppression of IL-1β secretion by nano-silica. Furthermore, we demonstrated that IL-10 induced by IFNT is essential to suppress IL-1β secretion in human macrophages because anti-IL-10 neutralizing antibody treatment with IFNT completely eliminated the inhibitory action of IFNT. Indeed, we recently showed that treatment with recombinant IL-10 inhibited nano-silica-induced IL-1β secretion in human placental cells and murine macrophages in a dose-dependent manner [25].

Similar to the actin-disrupting agent cytochalasin D, IFNT significantly decreased the uptake of nano-silica particles, indicating an inhibitory effect on phagocytosis. It is accepted that macrophages play important roles in the clearance of various environmental particles and microorganisms through SRs [27]. Accordingly, we found that IFNT decreased the expression of the class A SR MARCO but had no effect on the expression of other class A SRs such as MSR1 and OLR1, and class B SRs such as CD36 and SR-B1, suggesting that inhibition of MARCO expression is one of key events to suppress the nano-silica uptake and subsequent IL-1β secretion. To clarify the role of MARCO, we used siRNA strategy to knockdown MARCO in THP-1 macrophages. Although siRNA transfection achieved approximately 50% knockdown of MARCO mRNA, we could not detect a significant inhibition of nano-silica phagocytosis and IL-1β secretion. Kanno et al. [27] recently reported that MARCO transfection significantly increased the uptake of nano-size particles in macrophages, concluding that MARCO predominatly contributes to the rapid uptake of nanoparticles. Interestingly, Mukhopadhyay et al. [32] recently showed that MARCO not only enhances bacterial clearance by phagocytosis but also mediates silica particle-induced activation of NLRP3 inflammasomes in macrophages. In agreement, our present results showed that the partial knockdown of MARCO tended to suppress nano-silica-induced IL-1β secretion. Thus, further investigations using MARCO deficient macrophages are needed to clarify the role of MARCO in IFNT-suppressed IL-1β secretion induced by nano-silica.

Numerous studies on NLRP3 inflammasomes show its involvement in both pathogen-associated and sterile inflammatory diseases [9], [10], [11]. We and other investigators previously demonstrated the importance of NLRP3 inflammasomes in gout, type 2 diabetes mellitus, metabolic syndrome, atherosclerosis, myocardial infarction, asbestosis, silicosis, and Alzheimer's disease [9], [10], [11], suggesting that NLRP3 inflammasomes are a potential therapeutic target. Thus, IFNT may be safer and more effective compared with other type I IFNs, because higher doses of IFNT are needed to induce toxicity despite the similar potency between IFNT and other IFNs [7]. Soos et al. [6] also reported similar effectiveness of IFNT and IFNB as treatments that ameliorate the progression of murine EAE without causing toxicity. In addition, bovine IFNT has similar antiviral activity compared with that of IFNA, but less toxicity [33]. Accordingly, our results showed that IFNT inhibited IL-1β secretion to the same extent as IFNB. Importantly, Nakajima and Sokawa [34] reported no antibody production following oral administration of bovine IFNT, which is a foreign antigen in humans. Thus, IFNT may be an alternative therapeutic agent to IFNA and IFNB for the treatment for various inflammatory diseases.

Several limitations in this study should be noted. First, although IFNT inhibited both nano-silica-increased F-actin levels and phagocytosis, IFNT alone increased basal levels of F-actin, suggesting more complex relationship between F-actin levels and phagocytosis. In agreement, Frausto-Del-Rio et al. [35] recently demonstrated that IFN-γ alone increased basal levels of F-actin by activating Rac-1, leading to decreased phagocytosis of IgG-opsonized sheep red blood cells in human macrophages. Importantly, in this study, no additional responses to the chemokine CCL5 were detected in IFN-γ-treated macrophages, suggesting that F-actin levels may not be correlated with phagocytosis under certain conditions. Second, recent investigations demonstrated that type I IFN induces caspase-11 expression and activation, and this synergizes with NLRP3 inflammasome pathway to induce caspase-1-dependent IL-1β secretion [11], [22]. Therefore, we assume that contribution of type I IFN to the activation of NLRP3 inflammasomes may presumably vary depending on the experimental conditions. Thus, further investigations are required to elucidate the precise mechanisms underlying the inhibitory effects of IFNT on nano-silica-induced IL-1β secretion.

In conclusion, ruminant IFNT inhibits NLRP3 inflammasome-driven IL-1β secretion in human macrophages via multiple pathways, including the uptake of nano-silica particles, induction of pro-IL-1β, generation of ROS, and production of the anti-inflammatory cytokine IL-10. Several clinical trials are currently being conducted to determine the efficacy and tolerability of IFNT in patients with MS [8]. Thus, IFNT may not only be an alternative to IFNA and IFNB but also have therapeutic potential in the treatment of inflammatory disorders.

## Supporting Information

Figure S1 **Effects of rbIFNT and rhIFNB on STAT3 phosphorylation.** THP-1 macrophages were incubated with rbIFNT or rhIFNB for the indicated periods. Protein levels of STAT3 and phospho-STAT3 (p-STAT3) in cell lysates were detected by Western blot analyses. Representative photographs are shown.
(TIF)

Figure S2 **Effects of rbIFNT on NLRP3 inflammasomes.** (A) THP-1 macrophages were incubated for 48 h with rbIFNT or rhIFNB at the indicated concentrations. After priming with LPS (100 ng/mL) for 3 h, cells were treated with ATP (2 mM) for 6 h. IL-1β levels in supernatants were then determined using ELISA. (B) Mouse peritoneal cavity macrophages were isolated and incubated for 48 h with rbIFNT or rhIFNB at the indicated concentrations. After priming with LPS (100 ng/mL) for 3 h, cells were treated with nano-silica particles (100 µg/mL) for 6 h. IL-1β levels in supernatants were then determined using ELISA. (C–E) THP-1 macrophages were incubated for 48 h with or without rbIFNT or rhIFNB at the indicated concentrations. Expression of IL-1β, NLRP3, ASC, and caspase-1 mRNA was analyzed by real-time RT-PCR. GAPDH was used as an internal control. Data are expressed as means ±SEM (n = 3). (F) THP-1 macrophages were incubated for 48 h with or without rbIFNT (27 IU/mL). After priming with LPS (100 ng/mL) for 3 h, cells were treated with nano-silica particles (100 µg/mL) for 6 h. Protein levels of caspase-1 in cell lysates were detected by Western blot analyses. Representative photographs are shown.
(TIF)

Figure S3 **Effects of rbIFNT on IL-1β and IL-1α mRNA expression.** THP-1 macrophages were incubated for 48 h with rbIFNT. After priming with LPS (100 ng/mL) for 3 h or Pam3CSK4 (300 ng/mL) for 10 h, cells were treated with nano-silica particles (100 µg/mL) for 6 h. Subsequently, total RNA was extracted and analyzed by real-time RT-PCR for expression of IL-1β and IL-1α mRNA. Data are expressed as means ±SEM (n = 3); Significant differences were identified using ANOVA; ** p<0.01.
(TIF)
