# Supplementary figures and images for: Interferon-Tau Attenuates Uptake of Nanoparticles and Secretion of Interleukin-1β in Macrophages
Source: PLoS One. 2014 Dec 8;9(12):e113974. doi: 10.1371/journal.pone.0113974 (PMC4259327; doi:10.1371/journal.pone.0113974)

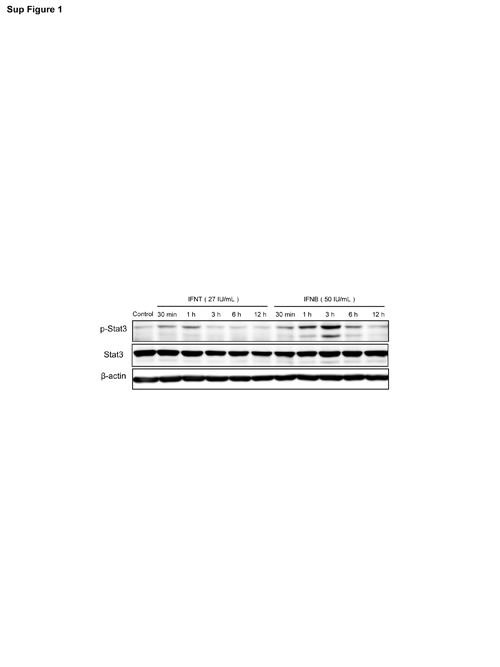

Supplement: Figure S1 — Effects of rbIFNT and rhIFNB on STAT3 phosphorylation. THP-1 macrophages were incubated with rbIFNT or rhIFNB for the indicated periods. Protein levels of STAT3 and phospho-STAT3 (p-STAT3) in cell lysates were detected by Western blot analyses. Representative photographs are shown. (TIF) [file pone.0113974.s001.tif]

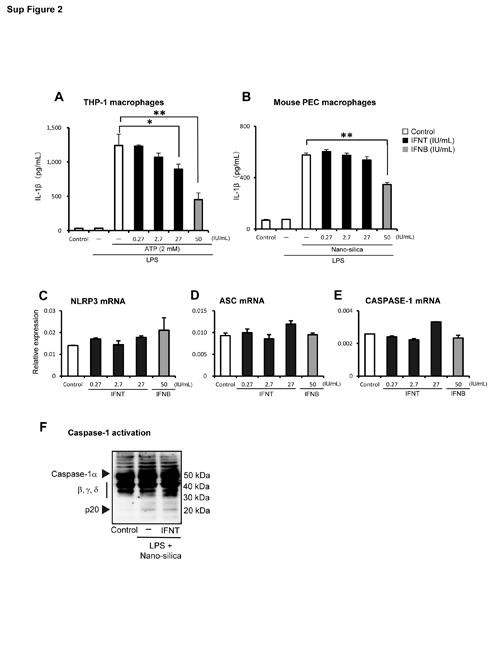

Supplement: Figure S2 — Effects of rbIFNT on NLRP3 inflammasomes. (A) THP-1 macrophages were incubated for 48 h with rbIFNT or rhIFNB at the indicated concentrations. After priming with LPS (100 ng/mL) for 3 h, cells were treated with ATP (2 mM) for 6 h. IL-1β levels in supernatants were then determined using ELISA. (B) Mouse peritoneal cavity macrophages were isolated and incubated for 48 h with rbIFNT or rhIFNB at the indicated concentrations. After priming with LPS (100 ng/mL) for 3 h, cells were treated with nano-silica particles (100 µg/mL) for 6 h. IL-1β levels in supernatants were then determined using ELISA. (C–E) THP-1 macrophages were incubated for 48 h with or without rbIFNT or rhIFNB at the indicated concentrations. Expression of IL-1β, NLRP3, ASC, and caspase-1 mRNA was analyzed by real-time RT-PCR. GAPDH was used as an internal control. Data are expressed as means ±SEM (n = 3). (F) THP-1 macrophages were incubated for 48 h with or without rbIFNT (27 IU/mL). After priming with LPS (100 ng/mL) for 3 h, cells were treated with nano-silica particles (100 µg/mL) for 6 h. Protein levels of caspase-1 in cell lysates were detected by Western blot analyses. Representative photographs are shown. (TIF) [file pone.0113974.s002.tif]

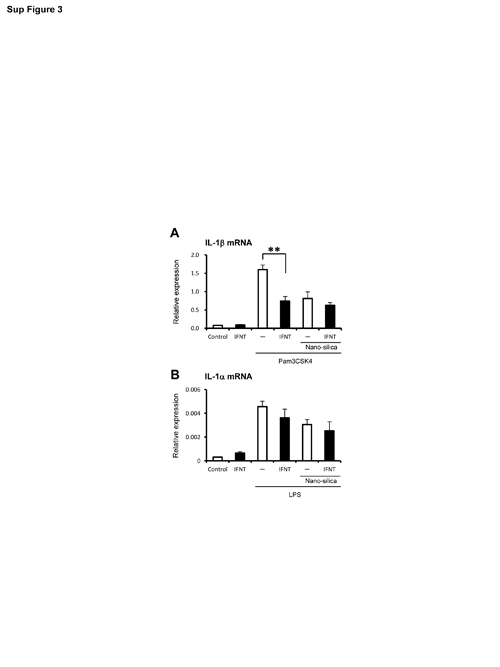

Supplement: Figure S3 — Effects of rbIFNT on IL-1β and IL-1α mRNA expression. THP-1 macrophages were incubated for 48 h with rbIFNT. After priming with LPS (100 ng/mL) for 3 h or Pam3CSK4 (300 ng/mL) for 10 h, cells were treated with nano-silica particles (100 µg/mL) for 6 h. Subsequently, total RNA was extracted and analyzed by real-time RT-PCR for expression of IL-1β and IL-1α mRNA. Data are expressed as means ±SEM (n = 3); Significant differences were identified using ANOVA; ** p<0.01. (TIF) [file pone.0113974.s003.tif]
